# Robust Angiogenesis and Arteriogenesis in the Skin of Diabetic Mice by Transient Delivery of Engineered VEGF and PDGF-BB Proteins in Fibrin Hydrogels

**DOI:** 10.3389/fbioe.2021.688467

**Published:** 2021-07-01

**Authors:** Alessandro Certelli, Paolo Valente, Andrea Uccelli, Andrea Grosso, Nunzia Di Maggio, Rosalinda D’Amico, Priscilla S. Briquez, Jeffrey A. Hubbell, Thomas Wolff, Lorenz Gürke, Edin Mujagic, Roberto Gianni-Barrera, Andrea Banfi

**Affiliations:** ^1^Cell and Gene Therapy, Department of Biomedicine, University Hospital of Basel, University of Basel, Basel, Switzerland; ^2^Vascular Surgery, Department of Surgery, University Hospital of Basel, University of Basel, Basel, Switzerland; ^3^Pritzker School of Molecular Engineering, University of Chicago, Chicago, IL, United States

**Keywords:** diabetes, angiogenesis, arteriogenesis, skin, VEGF, PDGF-BB, fibrin

## Abstract

Non-healing ulcers are a serious complication of diabetes mellitus and a major unmet medical need. A major cause for the lack of healing is the impairment of spontaneous vascularization in the skin, despite mostly normal blood flow in deeper large vessels. Therefore, pro-angiogenic treatments are needed to increase therapeutic perfusion by recruiting new arterial connections (therapeutic arteriogenesis). Vascular endothelial growth factor (VEGF) is the master regulator of angiogenesis in physiology and disease, but exploitation of its therapeutic potential requires careful control of its dose distribution in tissue. Co-delivery of platelet derived growth factor-BB (PDGF-BB) has been shown to expand the therapeutic window of VEGF and also improve associated arteriogenesis. We used a highly controlled protein delivery system, based on a clinically applicable fibrin-based platform, to investigate the angiogenic and arteriogenic potential of engineered versions (TG-) of VEGF and PDGF-BB proteins in the skin of diabetic and obese db/db mice. Intradermal delivery of therapeutically relevant doses of TG-VEGF and TG-PDGF-BB induced robust growth of new microvascular networks with similar efficacy as in normal littermate control mice. Further, TG-PDGF-BB prevented the formation of aberrant vascular enlargements by high TG-VEGF levels. As fibrin was degraded after the first week, the induced angiogenesis mostly regressed by 4 weeks, but it promoted effective arteriogenesis in the dermal layer. Therefore, controlled co-delivery of TG-VEGF and TG-PDGF-BB recombinant proteins is effective to induce angiogenesis and arteriogenesis in diabetic mouse skin and should be further investigated to promote diabetic wound healing.

## Introduction

Diabetes is a global health issue, ranking among the top 10 causes of death globally, and obesity-associated type 2 diabetes is the most prevalent form (Saeedi et al., 2019). Diabetic foot ulcers (DFU) are a common secondary complication of diabetes and are caused by inadequate arterial blood flow and/or sensory neuropathy (Tecilazich et al., 2011). Angiogenesis plays a significant role during the dynamic process of wound healing. In fact, DFU show areas of local skin ischemia with severely impaired blood flow (Falanga, 2005; Sharma et al., 2020). Therefore, therapeutic angiogenesis, which aims at stimulating neovascularization through local delivery of angiogenic factors to improve the perfusion of ischemic tissue, is an attractive treatment strategy.

Vascular endothelial growth factor-A (VEGF) is the master regulator of vascular growth and is the major molecular target for therapeutic angiogenesis (Uccelli et al., 2019; Gianni-Barrera et al., 2020).

Vascular endothelial growth factor’s therapeutic potential depends on its dose: uncontrolled and sustained VEGF expression can cause the growth of aberrant angioma-like vascular structures in both normal and ischemic tissues (Uccelli et al., 2019; Gianni-Barrera et al., 2020). However, the aberrant vascular growth by excessive VEGF pro-angiogenic stimulation can be prevented by promoting the recruitment of pericytes (Banfi et al., 2012; Gianni-Barrera et al., 2018), which are responsible for the maturation of the newly induced micro-vessels through both secreted signals, such as angiopoietins and TGFβ, and cell-to-cell contact (Armulik et al., 2011). Pericyte recruitment is mediated by Platelet-Derived Growth Factor-BB (PDGF-BB) (Banfi et al., 2012; Gianni-Barrera et al., 2018), which binds to PDGFR-β expressed on the surface of pericytes (Armulik et al., 2011). PDGF-BB over-expression and increased pericyte recruitment were found to limit excessive tumor angiogenesis by inhibiting endothelial proliferation (McCarty et al., 2007). Exceedingly high and sustained levels of PDGF-BB over-expression could occasionally induce transformation of endogenous mesenchymal cells (Marushima et al., 2020), pointing out the need to ensure a limited duration of treatment. On the other hand, we have previously shown that over-expression of PDGF-BB alone is incapable of starting the angiogenic process *in vivo* in the absence of VEGF (Banfi et al., 2012; Gianni-Barrera et al., 2016). However, in the context of VEGF co-delivery, balanced co-expression of VEGF and PDGF-BB in mouse skeletal muscle has been shown to: (a) ensure only normal angiogenesis despite high VEGF levels; (b) limit the size of abnormal vascular enlargements induced by VEGF through a reduction in endothelial proliferation; and (c) improve blood flow and stimulate the growth of arterioles (arteriogenesis) compared to VEGF alone in a mouse model of hindlimb ischemia (Banfi et al., 2012; Gianni-Barrera et al., 2018).

BKS.Cg-Dock7^m^ + / + Lepr^db^/J mice (herein called db/db mice), which are genetically deficient in the leptin receptor and spontaneously develop insulin resistance, diabetes and morbid obesity, represent the gold-standard model for type 2 diabetes mellitus (DM2) and are extensively used to study diabetes complications (Sullivan et al., 2004). Spontaneous reparative angiogenesis is severely impaired in diabetic patients (Rivard et al., 1999; Tamarat et al., 2003) and this defect is reproduced in db/db mice (Emanueli et al., 2007).

The use of recombinant protein factors is preferable to gene therapy vectors in clinical applications, in order to avoid heterogeneous and difficult-to-control levels of expression in the target tissue, possible immune reactions and safety concerns related to the transfer of genetic information (Martino et al., 2014). Since recombinant growth factors typically have a very short half-life *in vivo*, we have previously optimized a fibrin-based platform to deliver specific and homogeneous amounts of growth factors with a controlled duration, and we have shown it can be used with engineered VEGF to induce controlled and therapeutic angiogenesis in skeletal muscle (Sacchi et al., 2014). This platform provides a unique and highly standardized tool to precisely control the microenvironmental distribution of growth factor doses *in vivo*. Here we investigated this optimized fibrin-based platform to determine whether the intradermal co-delivery of engineered VEGF and PDGF-BB proteins can induce robust angiogenesis and increase the formation of feeding arteries (arteriogenesis) in the skin of db/db mice.

## Materials and Methods

### BKS.Cg-Dock7^m^+/+Lepr^db^/J (db/db) Mice

Mice were purchased from the Jackson Laboratory (Bar Harbor, ME, United States; JAX stock #000642) for further breeding. This murine strain carries a spontaneous point mutation in the gene encoding the leptin receptor, resulting in a protein with a truncated cytoplasmic domain that is functionally inactive (Chen et al., 1996) and therefore impairs the proper signal transduction (Ghilardi et al., 1996). It is a well validated animal model for the study of common metabolic abnormalities like obesity, dyslipidemia, and type 2 diabetes (Kobayashi et al., 2000; King and Bowe, 2016). Since both males and females homozygous for *Lepr*^db^ are sterile, the closely linked coat color mutation *misty* (*m*), which causes the generation of a gray coat instead of the usual black one, has been incorporated into animal stocks for maintenance of the *db* mutation. Breeding was performed by mating repulsion double heterozygotes, *Dock7*^m^+/+*Lepr*^db^, which following the Mendelian ratio, yields 25% diabetics (db/db: black, obese at weaning), 50% wild-type repulsion double heterozygotes (db/+: black, lean), and 25% misty wild type mice (wt: gray, lean). Blood glucose levels and body weights were systematically measured once per week from the age of 8 weeks till 10 weeks, at which time both male and female animals entered the experiment in similar ratio, in accordance with Swiss Federal guidelines for animal welfare (animal permit 2952, March 2018, by the Veterinary Office of the Canton of Basel-Stadt, Switzerland). Blood samples were collected from the tail vein of mice anesthetized with 3% isoflurane inhalation by using a syringe with a 29^1^/_2_G needle. Blood glucose levels were measured with a glucose meter (Ascensia Diabetes, Basel, Switzerland).

### Recombinant Murine TG-VEGF_164_ and TG-PDGF-BB Production

Recombinant mouse TG-VEGF_164_ and mouse TG-PDGF-BB were produced as previously described (Sacchi et al., 2014). Briefly, VEGF and PDGF-BB were engineered to contain at their N-terminus portion the substrate sequence for the transglutaminase (TG) factor XIIIa, derived from α_2_-plasmin inhibitor (α_2–_PI_1–8_ octapeptide: NQEQVSPL), which allows the covalent cross-linking of the modified factors into fibrin hydrogels and their release by enzymatic cleavage only. Fusion proteins were expressed into the Escherichia coli strain BL21 (Dε3) pLys (Novagen, Madison, WI, United States) and isolated as previously described (Zisch et al., 2001; Sacchi et al., 2014). Once produced, TG-VEGF and TG-PDGF-BB dimers were verified to be >99% pure by SDS/PAGE. Endotoxin level was verified to be under 0.05 EU/mg of protein using the human embryonic kidney (HEK)-Blue mTLR4 assay (Invivogen, San Diego, CA, United States).

### Fibrin Gel Preparation

Fibrin matrices were prepared by mixing human fibrinogen (plasminogen-, von Willebrand Factor-, and fibronectin-depleted; 25 mg/mL; Enzymes Research Laboratories, IN, United States), fluorescent Alexa 647-conjugated fibrinogen (0.5 mg/mL; Invitrogen, CA, United States), factor XIIIa (3 U/mL; CSL Behring, PA, United States), and thrombin (6 U/mL; Sigma-Aldrich, MO, United States) with 2.5 mM Ca^2+^ in 4-(2-hydroxyethyl)-1-piperazineethanesulfonic acid (Hepes) (Lonza, Basel, CH). Matrices containing TG-VEGF and TG-PDG-BB were obtained by adding the engineered proteins to the cross-linking enzymes solution before mixing with fibrinogen.

### Intradermal Fibrin Gel Implantation

**FIGURE 1:**
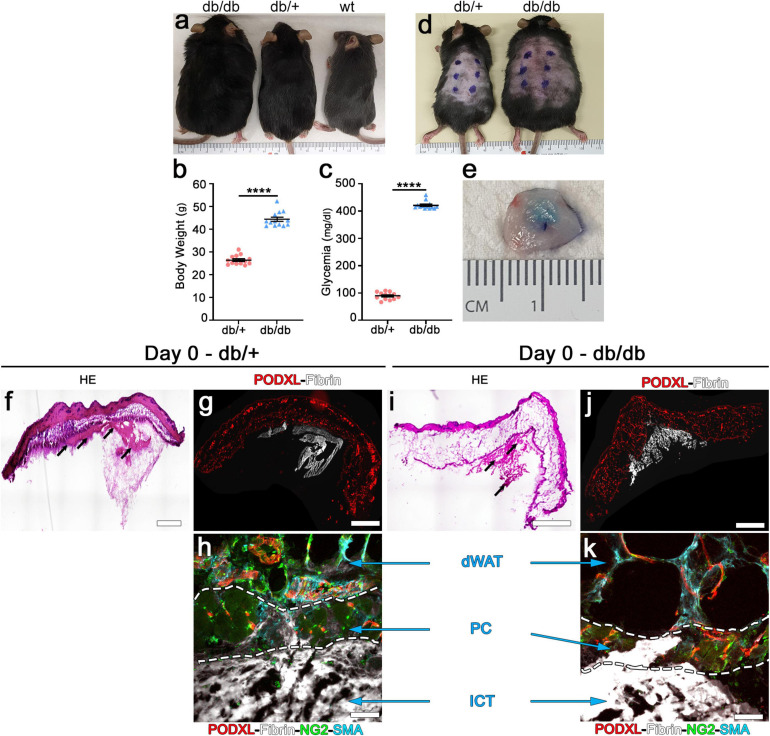
Intradermal injection and retention of fibrin gels in the skin of db mice. **(a)** Phenotype of diabetic db/db (left), heterozygous db/+ (center) and wild-type (wt, right) mice at 10 weeks of age. **(b)** Body weight measurements and **(c)** blood glucose levels of db/db diabetic mice and heterozygous db/+ littermates used as control for all the studies. Data represent mean values ± SEM. ****p < 0.0001 by the Mann–Whitney test; *n* = 13 animals per group. **(d)** Location of the intradermal implantations of fibrin gels in the back skin of heterozygous db/+ (left) and diabetic db/db (right) mice at 10 weeks of age (6 injections/mouse). **(e)** Fluorescent fibrinogen is visibly retained in the dermal skin layer after injection (Day 0). **(f,i)** H&E staining of skin cryosections showing the location of the fibrin gels (black arrows) after injection in heterozygous **(f)** and diabetic **(i)** mice. **(g,h,j,k)** Immunofluorescence staining for endothelium (podocalyxin, red), fluorescent fibrinogen (white), pericytes (NG2, green), and smooth muscle cells (α-SMA, cyan). **(h,k)** The injected fibrin gel is localized to the interstitial connective tissue (ICT) of dermis, under the dermal adipose white tissue (dWAT), and panniculus carnosus muscle (PC).

Animals were treated in accordance with Swiss Federal guidelines for animal welfare (animal permit 2952, March 2018), and the study protocol was approved by the Veterinary Office of the Canton of Basel-Stadt (Basel, Switzerland). A liquid volume of 20 μL fibrin hydrogels, which carry a mixture of 100 μg/mL TG-VEGF and 10 μg/mL TG-PDGF-BB, 100 μg/mL TG-VEGF alone or no factors (control), was aspirated rapidly with a 0.3-mL insulin syringe with integrated 29^1^/_2_G needle (Becton Dickinson) and injected into the dermal layers of 10-week old of either homozygous db/db, or heterozygous db/+ littermate control mice anesthetized with 3% isoflurane inhalation. A total of six individual injections were performed in each animal (Figure 1d) and each was retrieved and processed independently for histological analyses.

### Tissue Staining and Confocal Microscopy

All the studies were performed on frozen tissue sections. Briefly, mice were anesthetized and the tissues were fixed by vascular perfusion of 1% paraformaldehyde in PBS pH 7.4 for 4 min under 120 mm/Hg of pressure. Whole skin excisions of each individual injection site, which included the panniculus carnosus muscle and the interstitial connective tissue below it, were harvested, post-fixed in 0.5% paraformaldehyde in PBS for 2 h, cryo-protected in 30% sucrose in PBS overnight at 4°C, embedded in OCT compound (CellPath, Newtown, Powys, United Kingdom), frozen in freezing isopentane and cryosectioned. Tissue sections were then stained with H&E (25 μm sections) as previously described (Springer and Blau, 1997). Immunofluorescence on 25 μm-thick cross cryosections was performed as previously described (Ozawa et al., 2004). The following primary antibodies were used: goat polyclonal anti-mouse podocalyxin (2 μg/mL; R&D Systems, Minneapolis, MN, United States); mouse monoclonal anti-mouse α-SMA (0.2 μg/mL; clone 1A4, MP Biomedicals, Basel, Switzerland); rabbit polyclonal anti-NG2 (5 μg/mL; Merck Millipore, Darmstadt, Germany). Fluorescently labeled secondary antibodies (Invitrogen) were used at a final concentration of 10 μg/mL. Frozen sections were mounted with Faramount Aqueous Mounting Medium (Dako, Agilent Technologies, Basel, Switzerland), and fluorescence images were taken with 40x objectives on a Carl Zeiss LSM710 3-laser scanning confocal microscope (Carl Zeiss, Feldbach, Switzerland). Whole-tissue-slice fluorescence images were acquired with a 10x (numerical aperture, NA = 0.45) objective on Nikon Eclipse Ti2 microscope (Nikon, Egg/Zürich, Switzerland). Image analyses were performed with both LSM software Zen 2010 (Carl Zeiss, Feldbach, Switzerland) and Imaris 9.1.2 software (Bitplane, Zurich, Switzerland).

### Vessel Measurements

Vessel length density (VLD) and diameters were quantified in fluorescently immunostained cryosections as previously described (Ozawa et al., 2004). Briefly, VLD was measured in 5–8 randomly acquired fields in each area of effect per skin sample (*n* = 6–11 skin samples/group) by tracing the total length of vessels in the field and dividing it by the area of the field. Vessel diameters were measured in fluorescently immunostained sections as described (Ozawa et al., 2004). Briefly, 5–8 acquired fields in the areas of effect in each skin sample (*n* = 6–11 skin samples/group) were analyzed, measuring a total of 1,900 to 6,000 individual vessel diameters per time-point. The total vessel length was quantified by tracing the areas with a clear angiogenic effect in the whole skin section and multiplying it by its corresponding VLD value. The total angiogenic volume was estimated by multiplying the sum of the angiogenic areas in each consecutive histological section by the thickness of the sections. The number of arterioles was quantified in fluorescently immunostained cryosections. Arterioles were defined as vessels of regular shape and moderately larger than capillaries (15–30 μm) associated with a thick and homogeneous smooth muscle layer (positive for α-smooth muscle actin, α-SMA) coating the endothelial layer (positive for podocalyxin), as previously described (Springer et al., 2003; Gianni-Barrera et al., 2016). Arteriole density was quantified on 2 whole skin slices/sample, in the areas adjacent to the fields of induced angiogenesis (*n* = 6–9 skin samples/group). In control samples, where no angiogenic areas can be identified, arteriole density was quantified in areas of similar size and location as to those drawn in the treated samples. Fluorescence images were acquired as Z-Stack with 40x objective on a Carl Zeiss LSM710 3-laser scanning confocal microscope (Carl Zeiss, Feldbach, Switzerland). Three-dimensional immunofluorescence images were generated by using Imaris 9.1.2 software (Bitplane, Zurich, Switzerland). Whole-tissue-slice fluorescence images were acquired with a 10x (numerical aperture, NA = 0.45) or with a 20x (numerical aperture, NA = 0.75) objective on Nikon Eclipse Ti2 microscope (Nikon, Egg/Zürich, Switzerland). All image measurements were performed with CellSens software (Olympus, Volketswil, Switzerland) or with NIS-Elements Advanced Research software (Nikon, Egg/Zürich, Switzerland).

### Statistical Analysis

Data are presented as means ± standard error. The significance of differences was assessed with the GraphPad Prism 7.04 software (GraphPad Software). The normal distribution of all data sets was tested by D’Agostino and Pearson or Shapiro–Wilk. Multiple comparisons were performed with the parametric 1-way analysis of variance (ANOVA) followed by the Tukey or Sidak test for multiple comparisons, while single comparisons were analyzed with the non-parametric Mann–Whitney test or with the parametric *t*-test. *p* < 0.05 was considered statistically significant.

## Results

### Feasibility of Intradermal Fibrin Gel Implantation

We first sought to determine the feasibility of intradermal injections of fibrin gels in the dorsal skin of db/db mice. In fact, the liquid fibrinogen/enzymes mixture should not enter the subcutaneous space, where it would be rapidly lost in the virtual space over the body fascia, but rather be fully injected only in the thin dermal layers (intradermal injection) to ensure polymerization and retention. Homozygous db/db and heterozygous db/+ healthy littermate mice were identified based on their phenotype: black coat and obese for db/db and black coat and lean for db/+ (Figure 1a). Measurement of body weights and blood glucose levels of all animals which entered the experiments (male and female in about 1:1 ratio) confirmed that by the age of 10 weeks homozygous db/db mice were both obese and diabetic, whereas db/+ heterozygous littermate control mice had both physiologic weight and glycemia, as previously described (Hummel et al., 1966; Greer et al., 2006; Figure 1b,c; body weight: db/+ = 26.5 ± 0.6 g vs. db/db = 44.4 ± 0.9 g; glycemia: db/+ = 89.5 ± 3.7 mg/dl vs. db/db = 421.2 ± 4.1 mg/dl; *p* < 0.0001 for both comparisons).

Each animal received six injections of 20 μl in the skin of the back, labeled by external permanent marking (Figure 1d). To verify the anatomical localization of fibrin injections, animals were sacrificed after 2 h. All implanted gels were easily identified and found to be contained in the dermal layer in both db/+ and db/db mice, thanks to the labeling with fluorescent fibrinogen that also conferred a visible light blue color to the hydrogels (Figure 1e). Additionally, H&E staining (Figure 1f,i) and epifluorescence imaging of fluorescent fibrinogen, together with immunostaining for endothelium (podocalyxin), confirmed the intradermal location of the implanted gels and further showed a preferential localization in the interstitial connective tissue (ICT) below the *panniculus carnosus* (PC) muscle in both mouse strains (Figure 1g,h,j,k).

### TG-PDGF-BB Co-delivery Limits the Degree of Vascular Enlargement by High Levels of TG-VEGF in Diabetic Mouse Skin

**FIGURE 2:**
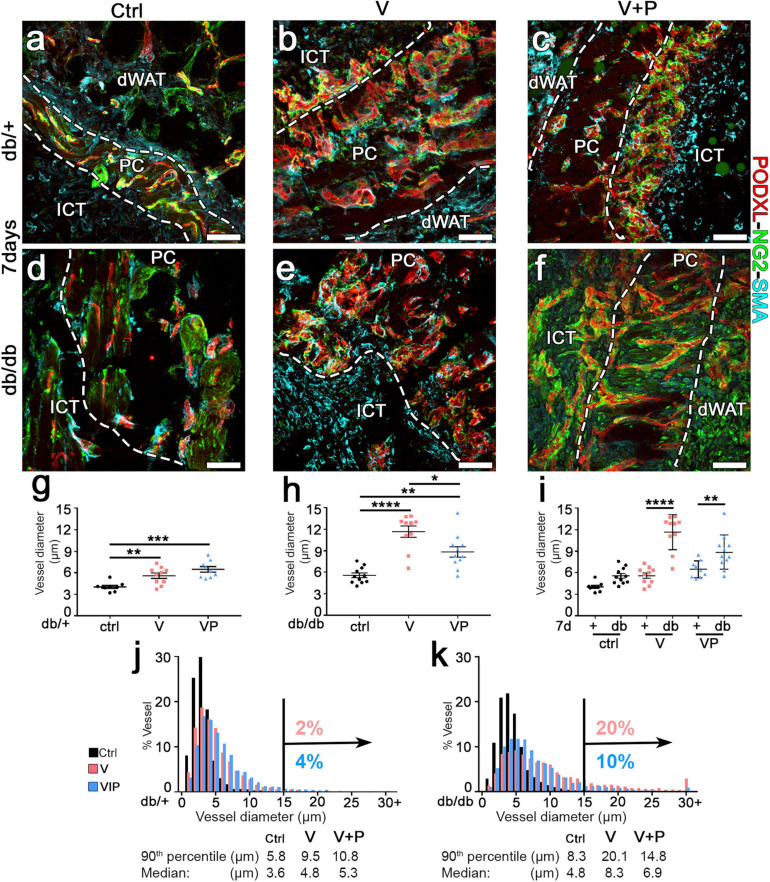
TG-PDGF-BB co-delivery limits the degree of vascular enlargement in diabetic skin. **(a–f)** Cryosections of dorsal skin of db/+ and db/db mice, harvested 7 days after implantation with fibrin gels decorated with TG-VEGF (V), TG-VEGF and TG-PDGF-BB (VP) or nothing (empty controls, ctrl), were immunostained for podocalyxin (endothelial cells, red), NG2 (pericytes, green), and α-SMA (smooth muscle cells, cyan); dWAT, dermal adipose white tissue; PC, panniculus carnosus; ICT, interstitial connective tissue. **(g–k)** Vessel diameters (in μm) were quantified in areas of fibrin implantation in db/+ (+) and db/db (db) mice. Results are shown as mean values of individual samples ± SEM (**g–i**; *n* = 9–11 samples/group) and as the distribution of vessel diameters over 1-μm intervals **(j,k)**. *p < 0.05, **p < 0.01, ***p < 0.001, ****p < 0.0001 by 1-way ANOVA with Sidak and Tukey multiple comparisons test. Scale bars = 50 μm in all panels.

The arteriogenic potential of VEGF and PDGF-BB co-delivery was previously found to be dose-dependent (Gianni-Barrera et al., 2016), i.e., it is optimally stimulated by higher doses of VEGF and PDGF-BB together. Therefore, fibrin gels were prepared with the maximum growth factor concentration that would not disrupt fibrin polymerization (Sacchi et al., 2014), i.e., 100 μg/mL of murine TG-VEGF alone or together with 10 μg/mL of murine TG-PDGF-BB. The ratio of PDGF-BB:VEGF of 1:10 was chosen based on a dose-dependent study of fibrin-based TG-VEGF and TG-PDGF-BB delivery to murine skeletal muscle (Sacchi et al., unpublished results), which showed that PDGF-BB:VEGF ratios between 1:3 and 1:20 were equally effective in ensuring normal angiogenesis, despite the highest possible VEGF dose cross-linked into the fibrin matrix. Seven days after implantation, control gels without any factor did not alter the pre-existing vasculature, which consisted of morphologically normal capillaries, closely associated with NG2+ pericytes (Figure 2a,d). Quantification of vessel diameters, analyzed either as average ± SEM (Figure 2g–i) or size distributions (Figure 2j,k), showed that pre-existing normal capillaries had a very homogeneous size and were uniformly distributed around a median diameter of 3.6 μm and 4.8 μm and with a 90th percentile value of 5.8 and 8.3 μm in db/+ and db/db mice, respectively (Figure 2j,k). In db/db mice TG-VEGF induced vascular structures that were associated with both NG2+ pericytes and α-SMA+ smooth muscle cells (Figure 2e) and were markedly enlarged compared to control vessels (average diameters: ctrl = 5.6 ± 0.3 μm, *V* = 11.7 ± 0.8; *p* < 0.0001; Figure 2h) with 20% of vessels larger than 15 μm (Figure 2k). In contrast, TG-PDGF-BB co-delivery induced mostly capillaries associated with NG2+/α-SMA- pericytes, similar to the capillaries found in control samples, both in db/+ and db/db skin (Figures 2c,f). TG-PDGF-BB also limited the degree of vascular enlargement induced by TG-VEGF, with only 10% of vessels larger than 15 μm vs. 20% for VEGF alone (Figure 2k) and an average diameter of 8.9 ± 0.7 μm (Figure 2h; *p* < 0.05 vs. VEGF = 11.7 ± 0.8 μm). The same high dose of VEGF delivered in heterozygous db/+ mice also induced morphologically normal capillaries associated with both pericytes and smooth muscle cells (Figure 2b). Vessels by TG-VEGF were slightly enlarged compared to controls (Figure 2g: average diameters: ctrl = 4.0 ± 0.2 μm, *V* = 5.6 ± 0.4; *p* < 0.01).

Interestingly, the size of angiogenic vessels induced by TG-VEGF in db/+ mice was markedly smaller compared to those found in db/db mice (Figure 2i; average diameters: V db/+ = 5.6 ± 0.4 vs. db/db = 11.7 ± 0.8 μm, *p* < 0000.1), with only 2% of vessels larger than 15 μm (Figure 2j) and similar to vessels found in animals treated with both TG-VEGF and TG-PDGF-BB (Figure 2g; average diameters: *V* = 5.6 ± 0.4; *p* < 0.01, VP = 6.5 ± 0.4; *p* = ns).

### TG-VEGF and TG-PDGF-BB Co-delivery Induces Robust Angiogenesis in Diabetic Mouse Skin

**FIGURE 3:**
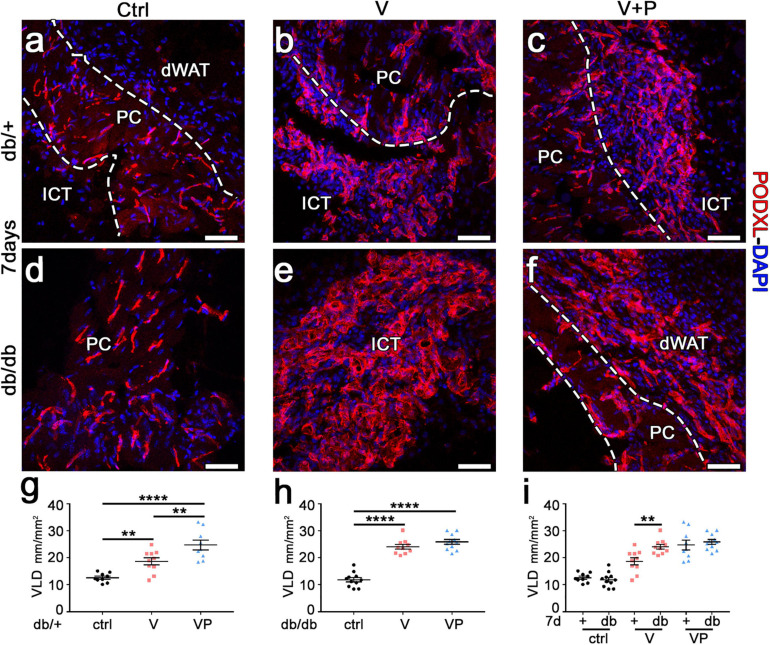
TG-VEGF and TG-PDGF-BB enable robust vessel formation in diabetic skin. **(a–f)** Cryosections of dorsal skin of db/+ (+) and db/db (db) mice, harvested 7 days after injection of V, VP, and control (ctrl) fibrin gels, were immunostained for podocalyxin (endothelial cells, red) and DAPI (nuclei, blue); dWAT, dermal adipose white tissue; PC, panniculus carnosus; ICT, interstitial connective tissue. **(g–i)** The amount of angiogenesis was quantified as VLD, vessel length density, expressed as millimeters of vessel length per square millimeter of area of effect (mm/mm^2^). Data represent mean values of each sample ± SEM (*n* = 9–11 samples/group). **p < 0.01, ****p < 0.0001 by 1-way ANOVA with Sidak and Tukey multiple comparisons test. Scale bars = 50 μm in all panels.

Diabetic db/db mice have been shown to display microangiopathy, e.g., in limb muscles, due to rarefaction of capillaries and arterioles, and also to have impaired reparative angiogenesis (Emanueli et al., 2007). Therefore, we investigated whether supra-physiologic doses of TG-VEGF alone or together with TG-PDGF-BB could overcome this deficit and induce robust vascular growth in the skin of diabetic mice. The amount of angiogenesis induced in the different conditions was quantified by measuring the vessel length density (VLD), defined as the total length of vessels in a given area independently of their diameter. TG-VEGF alone caused a significant increase in VLD compared to fibrin-only controls in both diabetic and normal mice (Figure 3a,b,d,e,g,h). Unexpectedly, the vascular density was actually higher in db/db mice compared to heterozygous mice (Figure 3e,b,i; V db/+ = 18.7 ± 1.3 vs. db/db = 24.1 ± 0.9 mm/mm^2^; *p* < 0.01). Vascular density in animals treated with TG-VEGF + TG-PDGF-BB was further increased compared to TG-VEGF alone in healthy db/+ mice (Figure 3b,c,g; *V* = 18.7 ± 1.3 and VP = 24.8 ± 1.8 mm/mm^2^; *p* < 0.01) and similar to the values obtained with both TG-VEGF alone or together with TG-PDGF-BB in db/db mice (Figure 3e,f,h,i).

**FIGURE 4:**
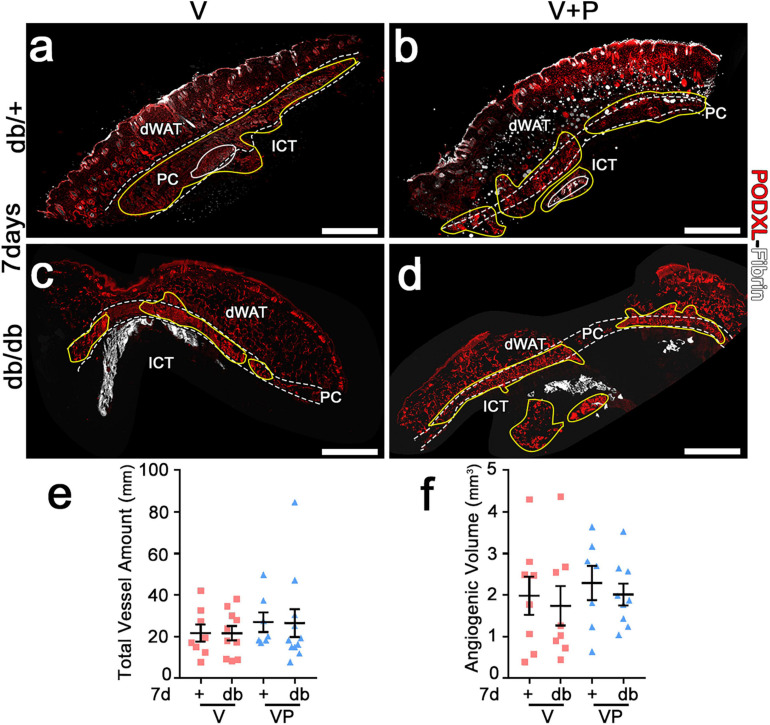
Both VEGF alone and with PDFG-BB enable similar amounts of vascular growth in diabetic skin as in healthy mice. **(a–d)** Immunostaining for podocalyxin (endothelial cells, red) and fibrin (white) was performed on cryosections of dorsal skin of normal heterozygous (db/+) and diabetic (db/db) mice 7 days after implantation with V and VP gels; dWAT, dermal adipose white tissue; PC, panniculus carnosus; ICT, interstitial connective tissue. The total vessel amount **(e)** and the total angiogenic volume **(f)** were quantified in all visible angiogenic areas (marked in yellow in **a–d**). Data represent mean values of each sample ± SEM (*n* = 8–11 samples/group). Scale bars = 1 mm in all panels.

The angiogenic effects of TG-VEGF and TG-VEGF + TG-PDGF-BB fibrin gels were induced over similarly large areas both in db/+ and db/db mice (Figure 4a–d). In fact, the total vessel amount (VLD × area of effect) in the implantation sites of fibrin gels was similar among both conditions and strains (Figure 4e: db/+ *V* = 21.6 ± 4.0 mm, VP = 26.9 ± 4.7 mm; db/db *V* = 21.6 ± 3.5 mm, VP = 26.4 ± 6.6 mm; *p* = ns for all comparisons; *n* = 8–11). An important parameter in view of a clinical application is the total angiogenic volume induced by a single injection. This was estimated by reconstructing the 3D volume of the angiogenic affect, i.e., adding all the angiogenic areas in each consecutive section and multiplying by the thickness of the sectioned tissue. The total angiogenic volume was also similar between both conditions and strains (Figure 4f: db/+ *V* = 2.0 ± 0.5 mm^3^, VP = 2.3 ± 0.4 mm^3^; db/db *V* = 1.7 ± 0.5 mm^3^, VP = 2.0 ± 0.3 mm^3^, *p* = ns for all comparisons, *n* = 8–9). Overall, these findings suggest that growth factor delivery at supra-physiologic doses stimulates angiogenesis in diabetic mice as efficiently as in healthy littermate controls.

### Vascular Growth Induced by Factor-Decorated Fibrin Hydrogels Is Transient in Diabetic Mouse Skin

It has been previously found in other tissues that newly induced vessels take about 4 weeks in order to form stable vascular networks, i.e., that would persist indefinitely and independently from the VEGF stimulus (Uccelli et al., 2019). Therefore, the newly formed vasculature induced by TG-VEGF alone or together with TG-PDGF-BB was studied 4 weeks after gel implantation. Since high-dose factor delivery was able to induce a robust angiogenic effect in db/db mice by 7 days, analyses were focused on diabetic animals.

**FIGURE 5:**
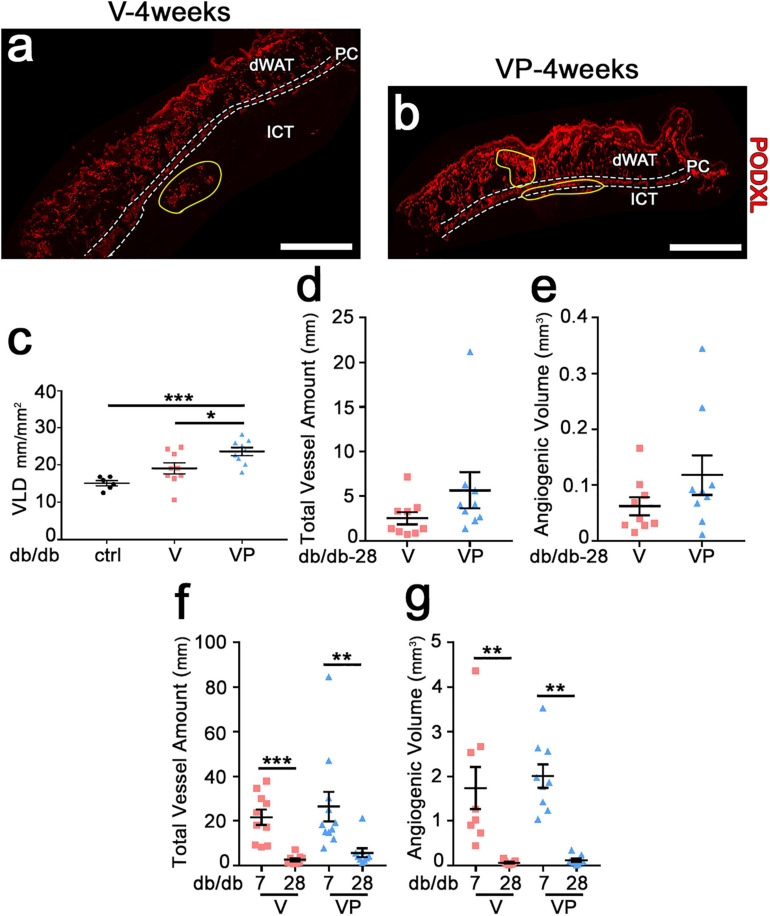
Newly induced angiogenesis is transient in diabetic skin. **(a,b)** Immunostaining with antibodies against podocalyxin (endothelial cells, red) was performed on cryosections of dorsal skin of diabetic mice 4 weeks after implantation of V, VP or control (ctrl) empty gels; dWAT, dermal adipose white tissue; PC, panniculus carnosus; ICT, interstitial connective tissue. Angiogenic areas are marked in yellow. **(c)** Vascular density in visible angiogenic areas was quantified by measuring VLD. Data are shown as mean values of each sample ± SEM (*n* = 6–9 samples/group); *p < 0.05, ***p < 0.001 by 1-way ANOVA with Tukey multiple comparisons test. **(d,f)** Total vessel amount in angiogenic areas after 4 weeks **(d)** and in comparison with the 7-day time-point **(f)**. **(e,g)** Total angiogenic volume after 4 weeks **(e)** and in comparison with the 7-day time-point **(g)**. Data represent mean values of each sample ± SEM (*n* = 8–11 samples/group). **p < 0.01, ***p < 0.001 by Kruskal–Wallis test. Scale bars = 1 mm in all panels.

Previous studies in skeletal muscle showed that fibrin hydrogels are completely degraded in about 10 days (Sacchi et al., 2014). In agreement with these findings, fibrin gels could not be detected in the skin of db/db mice after 4 weeks (Figure 5a,b). Further, visible angiogenic areas were greatly reduced compared to the 7-day time-point (yellow areas in Figure 5a,b). The vessel length density in the visible angiogenic areas in the TG-VEGF group was similar to controls (Figure 5c: *V* = 19.1 ± 1.5 mm/mm^2^ vs. control = 15.2 ± 0.7 mm/mm^2^, *p* = ns, *n* = 6–10), whereas in the presence of TG-PDGF-BB co-delivery it was still increased by about 35% compared with control samples (Figure 5c: VP = 23.6 ± 1.1 mm/mm^2^, *V* = 19.1 ± 1.5 mm/mm^2^ and control = 15.2 ± 0.7 mm/mm^2^, *p* < 0.05 and *p* < 0.001 for comparisons with VP, respectively, *n* = 6–11). The angiogenic effects of TG-PDGF-BB co-delivery were also still detectable over larger areas, though the difference was not statistically significant (Figure 5a,b: VP = 0.24 ± 0.09 mm^2^ vs. *V* = 0.13 ± 0.04 mm^2^, *p* = 0.13, *n* = 9 per group). Therefore, both the total vessel length (Figure 5d: *V* = 2.5 ± 0.7 mm, VP = 5.7 ± 2.0 mm, *p* = 0.05, *n* = 9 per group) and the total angiogenic volume (Figure 5e: *V* = 0.06 ± 0.02 mm^3^, VP = 0.12 ± 0.03 mm^3^, *p* = 0.17, *n* = 9 per group) in the implantation sites of TG-VEGF + TG-PDGF-BB fibrin gels were about 2-fold greater than those with TG-VEGF alone, though these differences were borderline significant. The comparison with the values measured after 7 days (Figure 5f,g) showed that most of the newly induced vessels had regressed by 4 weeks both with TG-VEGF alone and together with TG-PDGF-BB, though TG-PDGF-BB showed some benefit. In fact, quantification of total vessel amount showed a reduction of 88% for TG-VEGF alone and 78% for TG-VEGF + TG-PDGF-BB condition (Figure 5f: V-4 weeks = 2.5 ± 0.7 mm, V-7 days = 21.6 ± 3.5 mm, *p* < 0.001; VP-4 weeks = 5.7 ± 2 mm, VP-7 days = 26.4 ± 6.6 mm, *p* < 0.01). Quantifications of the total angiogenic volume showed an even more marked reduction of 96% with TG-VEGF alone and 94% with TG-PDGF-BB co-delivery (Figure 5g: V-4 weeks = 0.06 ± 0.02 mm^3^, V-7 days = 1.7 ± 0.5 mm^3^, *p* < 0.01; VP-4 weeks = 0.12 ± 0.03 mm^3^, VP-7 days = 2.0 ± 0.3 mm^3^, *p* < 0.01).

**FIGURE 6:**
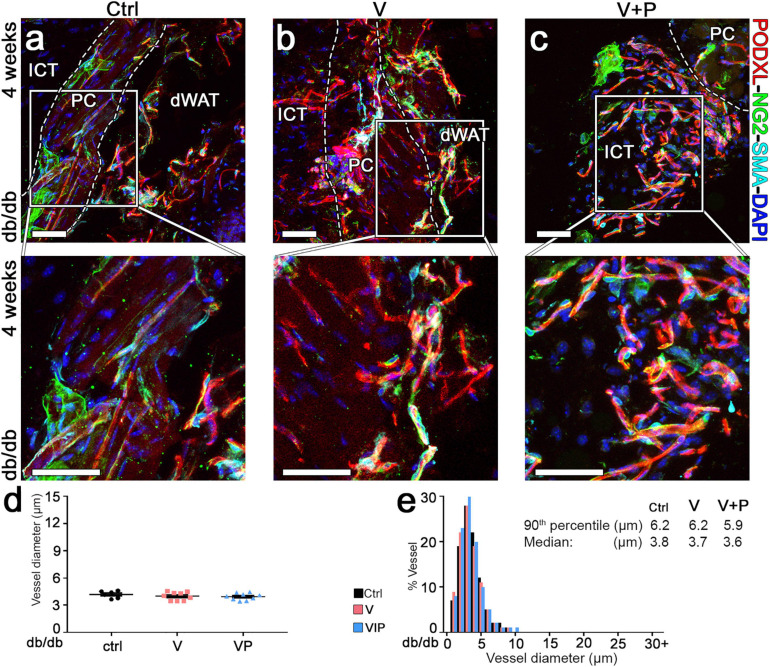
Long-term maturation and size of angiogenic vessels in diabetic skin. **(a–c)** Vascular morphology was analyzed 4 weeks after implantation of V, VP or control (ctrl) empty fibrin gels, by immunofluorescence staining for podocalyxin (endothelial cells, red), NG2 (pericytes, green), α-SMA (smooth muscle cells, cyan), and DAPI (nuclei, blue). The lower panels show higher-magnification views of the areas marked by the white squares in the top panels. Size bars = 50 μm in all panels; dWAT, dermal adipose white tissue; PC, panniculus carnosus; ICT, interstitial connective tissue. **(d,e)** Vessel diameters (in μm) were quantified and results are shown as mean values of each sample ± SEM **(d)** and as the distribution of vessel diameters over 1-μm intervals **(e)**; *n* = 6–9 samples/group.

The morphology of pre-existing vasculature in control samples was not altered by empty fibrin after 4 weeks (Figure 6a). None of the few remaining vessels induced by TG-VEGF evolved into aberrant structures (Figure 6b). All morphologically normal capillaries caused by TG-VEGF alone or with TG-PDGF-BB co-delivery were uniformly associated with NG2^+^ pericytes (Figure 6b,c), similarly to the normal pre-existing capillaries found in control samples (Figure 6a). Vessel diameters quantifications showed that pre-existing normal capillaries in areas implanted with control empty fibrin gels maintained the homogeneous sizes of the 7-day time point and were distributed around a median of 3.8 μm (Figure 6d,e). The vessels, which persisted under the TG-VEGF alone and with TG-PDGF-BB co-expression conditions, formed capillary-size micro-vascular networks with a homogeneous size distribution, similar to control samples (Figure 6d,e). Overall, these results suggest that transient delivery of both TG-VEGF alone and with TG-PDGF-BB induced a strong angiogenic effect in diabetic mouse skin by 7 days, which was transient and mostly regressed by 4 weeks.

### Co-delivery of TG-VEGF and TG-PDGF-BB Stimulates Arteriogenesis

**FIGURE 7:**
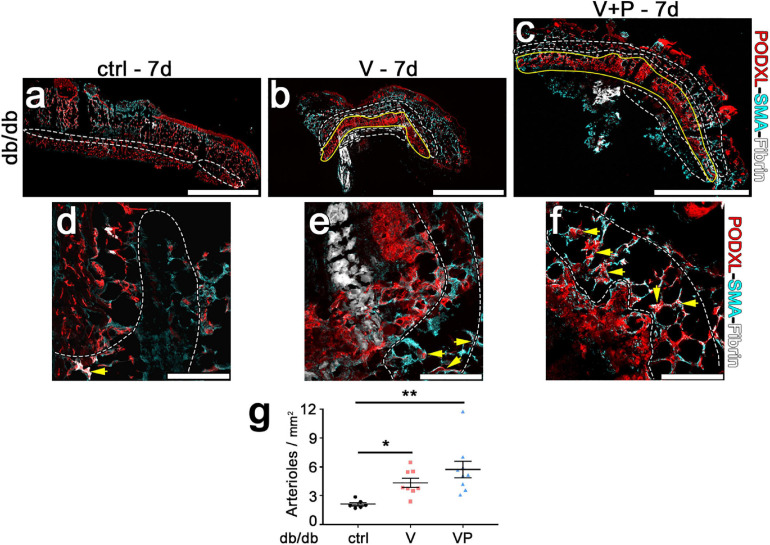
Efficient arteriole formation adjacent to areas of angiogenesis in diabetic skin. **(a–f)** Arterioles were identified by immunostaining 7 days after implantation of control fibrin (ctrl) or V and VP fibrin gels in peri-angiogenic areas (marked in white) surrounding the angiogenic areas (marked in yellow). Endothelium is stained in red (Podocalyxin) and smooth muscle cells in cyan (α-SMA). **(d–f)** Higher-magnification images of representative peri-angiogenic areas (in white). Yellow arrows indicate α-SMA-positive arterioles. Size bars = 2 mm **(a–c)** and 200 μm **(d–f)**. **(g)** Quantification of the density of arterioles adjacent to the angiogenic areas (number of arterioles/mm^2^ of area). Data are shown as mean values of each sample ± SEM (*n* = 6–9 samples/group); **p < 0.01, *p < 0.05 by Kruskal–Wallis test.

In order to be beneficial to ischemic tissues, therapeutic angiogenesis requires the growth of both microvascular capillaries, responsible for nutrient and gas exchanges, and larger caliber arteries and arterioles, capable of supplying increased blood flow to the microvascular networks in the ischemic regions. Therefore, we quantified arterioles in the skin of diabetic mice 7 days after injection of the factor-decorated fibrin gels, when a marked angiogenic effect was induced by both TG-VEGF and TG-VEGF + TG-PDGF-BB co-delivery (Figure 7). Arterioles were identified as regularly shaped vessels, larger than capillaries (diameter >15 μm) and covered by a homogeneous smooth-muscle coating (Springer et al., 2003; Gianni-Barrera et al., 2016). Arteriole density in the tissue immediately adjacent to the angiogenic areas was significantly increased by both treatments compared to fibrin-only controls (Figure 7e: VP = 5.7 ± 0.8 arterioles/mm^2^, *V* = 4.3 ± 0.5 arterioles/mm^2^ and control = 2.0 ± 0.2 arterioles/mm^2^, *p* < 0.01 and *p* < 0.05 vs. control, respectively; *n* = 6–9). These results suggest that both TG-VEGF alone and together with TG-PDGF-BB could induce organized vascular trees already in 7 days, comprised of both angiogenesis and arteriogenesis.

## Discussion

Here we found that intradermal delivery of fibrin hydrogels decorated with high levels of recombinant TG-VEGF protein could promote robust growth of new microvascular networks in the skin of diabetic mice with a similar efficacy as in non-diabetic controls. Further, TG-PDGF-BB co-delivery could modulate the angiogenic response and yield more physiological vascular networks. The duration of factor delivery in the tissue was limited to about 1 week by the natural process of fibrin degradation, and the resulting vascular growth was also transient, with most of the newly induced vessels regressing by 4 weeks. However, the kinetics of factor delivery was sufficient to generate both a robust expansion of intradermal microvascular networks and stimulate a 2- to 3-fold increase in feeding arteries around the angiogenic areas.

The monogenic diabetic db/db mouse is the gold-standard type 2 diabetic model. Although the specific autosomal recessive mutation in the leptin receptor is not usually present in human type 2 diabetes, this model reproduces many features of the human disease through the obesity-induced peripheral insulin resistance, which is an important pathogenic mechanism of human Type 2 diabetes (Grada et al., 2018). In particular, in diabetic patients reparative angiogenesis is impaired, due for example to a reduction in spontaneous VEGF signaling, changes in inflammation-related pathways and accumulation of advanced glycation end products (Rivard et al., 1999; Tamarat et al., 2003), and this defect is also reproduced in db/db mice (Emanueli et al., 2007). The results reported here show that controlled delivery of supra-physiological doses of recombinant TG-VEGF protein could be effective to overcome the diabetes-induced impairment of spontaneous reparative angiogenesis and, in particular, the combination with TG-PDGF-BB can limit undesired effects of the high VEGF dose, such as excessive vascular enlargement.

A lack of physiological maturation by pericyte association is an intrinsic and widely described property of vessels induced by high VEGF doses. For example, topical application of recombinant VEGF protein has been reported to accelerate wound healing in diabetic mice, but it also led to the formation of leaky malformed vasculature and edema (Galiano et al., 2004). Also, a phase I clinical trial has been performed using topical recombinant human VEGF (telbermin) on DFUs (Hanft et al., 2008). However, even though there were positive trends suggestive of potential signals of biological activity observed for incidence of complete ulcer healing and time to complete ulcer healing, clinical benefit has not been established (Okonkwo and DiPietro, 2017). Indeed, here we also found that the high levels of TG-VEGF in the fibrin hydrogel induced abnormally dilated vessels in the diabetic animals, whose pericyte coverage had been partially lost and substituted by α-SMA-positive smooth muscle cells, but PDGF-BB co-delivery prevented excessive vascular enlargement by high VEGF levels and restored physiological pericyte coverage, yielding normal networks of mature capillaries, similarly to the effects previously described in skeletal muscle (Gianni-Barrera et al., 2018).

A major complication of type 2 diabetes mellitus in humans is deficient wound repair arising in part from impaired angiogenesis. In fact, vascularization is crucial in supporting newly formed granulation tissue within the wound bed by delivering oxygen and nutrients (Gurtner et al., 2008). Similar to chronic ulcers in diabetic patients, db/db mice exhibit severe impairments in wound healing, such as delay in wound closure, decreased wound bed vascularity (Greenhalgh et al., 1990; Michaels et al., 2007) and decreased granulation tissue formation, which constitutes the principal process of wound repair (Martino et al., 2011). Therefore, the efficient induction of angiogenesis by intradermal co-delivery of VEGF and PDGF-BB could help granulation tissue formation and counteract the defect in VEGF expression and defective angiogenesis, which contribute to the healing impairment in diabetes (Altavilla et al., 2001).

Newly induced microvascular networks are susceptible to regression upon withdrawal of growth factors and it has been found that VEGF stimulation is required for 3–4 weeks for vessels to persist indefinitely (Lazarus and Keshet, 2011). Consistent with the fact that the factor-decorated fibrin hydrogels are degraded in about 10 days (17), here we found that most of the vasculature that was newly induced after 1 week had regressed by 4 weeks. Transient delivery of growth factors is desirable for safety, but regression of the induced angiogenesis might be a concern for efficacy. On the other hand, it should be considered that wound healing is also a transient process that physiologically takes place over 10–15 days (Martino et al., 2011). Therefore, the transient angiogenesis induced by TG-VEGF and TG-VEGF + TG-PDGF-BB by 7 days is consistent with the time-frame over which a therapeutic stimulation of vascular growth would be required to accelerate diabetic wound healing. However, in order to investigate the therapeutic potential of a longer persistence of induced microvascular networks on wound healing, this could be achieved by supplementing the hydrogels with optimized doses of TG-aprotinin (Lorentz et al., 2011), which modulates fibrin degradation by proteases and allows a full customization of the *in vivo* duration of factor release up to 4 weeks (Sacchi et al., 2014).

Characteristics of the microvascular impairment during wound healing in db/db mice include also decreased number and density of arterioles (Martin et al., 2003), which are required to promote functional improvement by supplying increased blood flow to the affected tissues (Schaper, 2009). VEGF is well known to favor arterial differentiation and to be required for adult arteriogenesis (Simons and Eichmann, 2015). In this respect, it was also recently shown that co-expression of VEGF and PDGF-BB sustained a dose-dependent increase in the amount of induced arterioles in non-ischemic murine skeletal muscle (Gianni-Barrera et al., 2016). In agreement with these findings, the transient and robust growth of new microvascular networks by high-doses of TG-VEGF in db/db skin was accompanied by the generation of numerous arterioles in the immediately adjacent dermal areas (Figure 7) and this arteriogenesis process could be further promoted by TG-PDGF-BB co-delivery by about 25%.

In conclusion, these data suggest that intradermal co-delivery of TG-VEGF- and TG-PDGF-BB-decorated fibrin hydrogels is a promising strategy to promote transient and robust angiogenesis in diabetic skin, accompanied by arteriogenesis, and support the further investigation of the therapeutic potential of this approach to accelerate the healing of chronic diabetic wounds.

## Data Availability Statement

The raw data supporting the conclusions of this article will be made available by the authors, without undue reservation.

## Ethics Statement

The animal study was reviewed and approved by the Veterinary Office of the Canton of Basel-Stadt (Basel, Switzerland).

## Author Contributions

AB, RG-B, EM, LG, TW, and JH conceived and designed the study. AC, PV, AU, AG, ND, RD’A, PB, RG-B, and AB performed the experiments, acquired, analyzed, or interpreted the data. RG-B and AC wrote the first draft of the manuscript. All authors contributed to manuscript revision, read, and approved the submitted version.

## Conflict of Interest

The fibrin gel immobilization scheme is the subject of patents upon which JH is named as inventor and has been licensed by a company in which JH is a shareholder. The remaining authors declare that the research was conducted in the absence of any commercial or financial relationships that could be construed as a potential conflict of interest.
